# eHealth Literacy and its Associated Factors Among Health Professionals During the COVID-19 Pandemic in Resource-Limited Settings: Cross-sectional Study

**DOI:** 10.2196/36206

**Published:** 2022-07-13

**Authors:** Getanew Aschalew Tesfa, Delelegn Emwodew Yehualashet, Helen Ali Ewune, Addisu Getnet Zemeskel, Mulugeta Hayelom Kalayou, Binyam Tariku Seboka

**Affiliations:** 1 School of Public Health College of Medicine and Health Science Dilla University Dilla Ethiopia; 2 Department of Midwifery College of Medicine and Health Science Dilla University Dilla Ethiopia; 3 Department of Health Informatics School of Public Health, College of Medicine and Health Sciences Wollo University Wollo Ethiopia

**Keywords:** eHealth literacy, COVID-19, health information, health literacy

## Abstract

**Background:**

The COVID-19 pandemic has wreaked havoc on health care systems and governments worldwide. Although eHealth literacy is acknowledged as a critical component of public health, it was overlooked during the pandemic. To assist patients and their families, health professionals should be knowledgeable about online health information resources and capable of evaluating relevant online information. In a resource-constrained situation, the level of eHealth literacy among health professionals is not well documented.

**Objective:**

The aim of this study was to assess the eHealth literacy level and its associated factors among health professionals working in Amhara regional state teaching hospitals, Ethiopia.

**Methods:**

A self-administered questionnaire was used in an institutional-based cross-sectional study design. Descriptive statistics were calculated to describe eHealth literacy statements and key variables using SPSS v.24. Bivariable and multivariable logistic regression models were fit to identify factors related to eHealth literacy. Variables with *P*<.05 were declared to be statistically significant predictors.

**Results:**

A total of 383 participants completed and returned the questionnaire with a response rate of 90.5%. Health professionals demonstrated a moderate level of eHealth literacy (mean 29.21). Most of the professionals were aware of the available health resources located on the internet, and know how to search and locate these resources. However, they lack the ability to distinguish high-quality health resources from low-quality resources. Factors that were significantly associated with eHealth literacy were computer access, computer knowledge, perceived ease of use, and perceived usefulness of eHealth information resources.

**Conclusions:**

It is crucial to provide training and support to health care workers on how to find, interpret, and, most importantly, evaluate the quality of health information found on the internet to improve their eHealth literacy level. Further research is needed to explore the role of eHealth literacy in mitigating pandemics in developing countries.

## Introduction

The internet is currently one of the most widely used tools for obtaining information about health care and medical conditions [1], providing health professionals with unprecedented access to a massive volume of relevant and high-quality current health care information [2]. Moreover, the internet has a significant impact on health and health care since it can improve health care delivery and help decision-making for health care workers [3]. For modern health care recipients, the internet provides a useful and accessible source of health-related information [4].

Health literacy is vital for people who interact with the digital world, with its diverse information and sources [5], and plays an important role in evaluating online health information [6], especially during pandemics such as COVID-19, the disease caused by infection with the novel coronavirus SARS-CoV-2, which has posed unprecedented challenges worldwide [7,8]. The COVID-19 pandemic has been accompanied by the rapid spread of disinformation or fake news via social media platforms and other outlets. The dissemination of this misinformation could lead to people acting inappropriately, thereby jeopardizing governments’ and health authorities’ efforts to manage the pandemic [9]. To control this worldwide health catastrophe, eHealth literacy is recommended among the interdisciplinary and multidimensional techniques [9]. Individuals with adequate eHealth literacy are more likely to utilize the internet to obtain health-related information and believe they are capable of applying web-based knowledge to improve their health [10].

eHealth literacy is defined as the ability to seek out, find, interpret, evaluate, and appraise health information from electronic sources, as well as apply that knowledge to address or solve a health problem. This composite skill necessitates the ability to interact with technology, think critically about media and science concerns, and navigate a large assortment of information tools and sources to obtain the information needed to make decisions [5].

The eHealth Literacy Scale (eHEALS) was developed in response to the need to assess eHealth literacy in a variety of populations and settings. The eHEALS is a self-report tool that can be administered by a health professional and is based on a person’s perception of their skills and knowledge in each of the measured domains. The test is intended to offer a broad estimate of consumer eHealth abilities that can be used to guide clinical decision-making and health-promotion planning with individuals or groups. The eHEALS has potential to be used in a clinical setting to identify individuals who may or may not benefit from referrals to an eHealth intervention or resource [1,5].

Controlling pandemics requires strong enabling environments as well as modern and digitized health information systems. Beyond the COVID-19 pandemic, it is critical to promote digital solutions [11]. Countries with health information systems that combine data from the health and long-term care sectors are likely to be better prepared to deal with this challenge [12]. The use of online consultations from hospitals and health care centers has been found to be a safe and effective way to mitigate the pandemic’s negative effects [13].

The outbreak of COVID-19 has made eHealth literacy more vital than ever [9]. However, this has been an underestimated issue during the pandemic [14]. eHealth literacy has a direct effect on health-promoting behaviors by improving health information–seeking behavior, which can ultimately lead to health-promoting behavior and health outcomes [15]. eHealth literacy has the potential to increase adherence to infection prevention and control measures, promote healthy habits, and maintain health care workers’ health. This would help contain the COVID-19 pandemic and further mitigate its impacts [16]. The world learned via COVID-19 that eHealth is not an optional or excessive approach to health care but rather a crucial, safer, and effective means of providing health care to both individuals with underlying problems and others during such periods. Because it is critical to obtain accurate information from reputable sources when self-managing diseases, individuals’ health perceptions and behaviors are negatively influenced by misinformation [17,18].

Aside from having a basic understanding of how to utilize the internet and eHealth literacy, health care practitioners should be knowledgeable on how to evaluate sources of information as reputable sources of information [19]. According to a study conducted at the University of Gondar (UOG) specialized hospital, patients have a poor level of eHealth literacy, implying that there is a need to bridge the skill gap [20]. This study could imply that this is where health care professionals can help patients make health-related decisions, specifically to assist patients and families in getting up-to-date, reliable, and quality health information, and identifying and analyzing suitable web sources for such decisions. However, health care practitioners must be eHealth literate to provide this assistance [21]. The potential predictor variables of this study were identified based on the review of findings from other related literature. Previous studies showed that variables such as age, sex, professional background, work experience, training about information retrieval techniques, computer knowledge, perception toward web resources, and computer accessibility were significantly associated with eHealth literacy [16,22-26].

The expansion of smartphone penetrations, the growing number of internet users, and information needs in developing nations are the most compelling reasons for assessing eHealth literacy and its associated factors among health care professionals to maximize eHealth benefits. However, there is limited information on eHealth literacy among health care workers in Ethiopia. Therefore, the aim of this study was to assess eHealth literacy among health professionals at Amhara regional state teaching hospitals, as well as to identify factors that influence eHealth literacy. We postulated the following three key hypotheses.

Hypothesis 1: computer knowledge positively correlates with eHealth literacy.

Hypothesis 2: there is a significant positive association between eHealth literacy and perception (perceived usefulness and perceived ease of use).

Hypothesis 3: The accessibility of computers is positively linked with eHealth literacy.

## Methods

### Study Area, Design, and Period

The study was conducted among health professionals working at Amhara regional state teaching hospitals in Ethiopia from February 23 to May 10, 2020, using an institutional-based cross-sectional study design. Ethiopia is divided into nine regions and two city administrations. The Amhara region is the country’s second-largest and most populous among these regions [27]. The Tibebe Ghion specialized teaching hospital in Bahir Dar city and UOG specialized teaching hospital in the town of Gondar are the two specialized teaching hospitals in the region. At the time of the survey, the UOG specialized teaching hospital had 1076 permanent employees, whereas the Tibebe Ghion specialized teaching hospital had 738 permanent employees.

### Study Procedure

The study’s sample size was calculated using the following single-population proportion formula by assuming that 50% of health professionals have a high degree of eHealth literacy and a 10% nonresponse rate.


[Za^2^×p(1–p)]/d^2^=[(1.96)^2^×0.5(1–0.5)]/0.05^2^=384.4+38.4=423

Where Za^2^ is the *Z* statistic (value of the standard normal distribution) at 95% confidence, d is the margin of error at 5%, and p is the single-population proportion.

Participants of the study were selected from the UOG and Tibebe Ghion specialized teaching hospitals. To guarantee a fair distribution of the entire sample, health care workers from each hospital were stratified based on profession and then study participants were chosen using a simple random sampling technique.

### Measures

#### eHealth Literacy

eHealth literacy was measured using the eHEALS, which was introduced by Norman and Skinner [5] to determine consumers’ combined knowledge and perceived skill in finding, evaluating, and applying eHealth information to health problems. The eHEALS has eight items that are scored on a 5-point Likert scale ranging from 1 (strongly disagree) to 5 (strongly agree), and the total score ranges between 5 and 40. This scale was reported as a reliable tool with a Cronbach α coefficient of .88 in the original study [5] and had a Cronbach α of .847 in this study. A high eHEALS score indicates a high eHealth literacy level, whereas a low eHEALS score indicates low eHealth literacy.

#### Computer Access

Computer access was measured by a yes-or-no question; health professionals were asked whether they can access computers in their working area/hospital or not.

#### Perceived Usefulness

Perceived usefulness was measured using five closed questions, and study participants who scored the mean and above on the 5-point Likert scale questions were categorized according to whether they considered that using eHealth information resources to be useful or not useful.

#### Perceived Ease of Use

Perceived ease of use refers to the degree to which a person believes that using eHealth information resources is free of effort, which was measured using four closed questions. Study participants who scored the mean and above on the 5-point Likert scale questions were categorized as to whether or not they considered that using eHealth information resources is easy.

#### Computer Knowledge

Computer knowledge was assessed based on participants’ self-perceived reports; respondents were asked to rate their level of computer knowledge according to five categories (none, beginner, below average, average, above average) with respect to basic computer skills and internet navigation. According to these responses, the participants were dichotomized as having poor or good computer knowledge for further analysis.

### Data Collection

Self-administered questionnaires were used to collect the data. The questionnaire included 29 questions divided into four sections: sociodemographic characteristics (six items), the eHEALS (eight items), perceived usefulness of eHealth information resources (five items), and perceived ease of use (four items).

Data collectors received training on data collection techniques, maintaining the confidentiality of health professionals’ information, obtaining informed consent, study participants’ rights, and all study protocols. Before the actual data collection, a pretest was performed among 5% of the entire sample population outside the study area, and any required corrections and revisions to the questionnaire were implemented. The completeness of the questionnaire was checked daily.

### Data Processing and Analysis

Epi-info version 7 was used for data entry. Data cleaning and coding were performed before any statistical analysis. Data analysis was performed with SPSS version 20 software. To describe the participant characteristics and study objectives, key variables are summarized in terms of descriptive frequencies and mean (SD).

Model fit was checked using the Hosmer and Lemeshow goodness-of-fit test. Bivariate analysis was then used to examine the relationship between individual independent variables and the dependent variable. Variables with *P*≤.20 in the bivariate analysis were then entered into a multivariable logistic regression model to examine the relationship between selected independent variables and the outcome variable. In the multivariate analysis, variables with *P*<.05 according to the odds ratio were determined to be statistically significant independent predictors.

### Ethical Statement

On behalf of the UOG College of Medicine and Health Sciences, ethical approval was secured from the Institute of Public Health (number IPH/840/02/2020). Written consent was obtained from each study participant. The data were collected anonymously and participants’ privacy was respected. Privacy of all of the information gathered was maintained and the data were solely utilized for research purposes.

## Results

### Characteristics of the Study Participants

With a response rate of 90.5%, 383 of the total disseminated questionnaires were returned. The majority of the 383 study participants were male (Table 1). The mean age of the participants was 28.3 (SD 3.37) years. Nurses accounted for the highest proportion of professionals, followed by medical doctors and midwives. Less than 30% of the participants had received information-retrieval training on eHealth information sources (Table 1).

**Table 1:** Sociodemographic characteristics of the study participants (N=383).

Variables		Participants, n (%)
**Sex**		
	Male	239 (62.4)
	Female	144 (37.6)
**Age group (years)**		
	20-24	18 (4.7)
	25-29	254 (66.3)
	≥30	111 (29.0)
**Professional background**		
	Nurse	158 (41.3)
	Medical doctor	94 (24.5)
	Pharmacist	30 (7.8)
	Midwife	54 (14.1)
	Laboratory	24 (6.3)
	Other	23 (6.0)
**Work experience (years)**		
	1-3	226 (59.0)
	4-6	119 (31.1)
	≥7	38 (9.9)
**Computer access**		
	No	166 (43.3)
	Yes	217 (56.7)
**Received training on information retrieval**		
	No	269 (70.2)
	Yes	114 (29.8)

### eHealth Literacy

The overall mean score for eHealth literacy was 29.21 (SD 7.08), which is considered to be moderate; among the 383 health professionals surveyed, 225 (58.7%) had high eHealth literacy and the other 41.3% (n=158) had low eHealth literacy, defined as those scoring above and below the mean on the eHEALS, respectively. As shown in Table 2, 240 (62.7%) of the total survey participants reported knowing what health resources are available on the internet and 235 (61.3%) of the 383 participants agreed with the statement “I know where to find helpful health resources on the internet.”

Only 221 (57.7%) of the 383 participants claimed that they could identify high-quality health resources from low-quality resources, indicating that almost half of the health professionals have trouble distinguishing quality health resources on the internet; 231 (60.3%) of the participants reported that they have the skills to evaluate health resources they found on the internet (Table 2).

Based on the participants’ background characteristics, the majority of both the male and female participants had a high level of eHealth literacy. By contrast, nearly half of the nurses and one-third of medical doctors had a lower level of eHealth literacy (Table 3).

**Table 2:** Distribution of eHealth Literacy Scale (eHEALS) responses (N=383).

eHEALS statements	Rating scale, n (%)						Mean score
	Strongly disagree	Disagree	Unsure	Agree	Strongly agree		
I know what health resources are available on the internet	7 (1.8)	47 (12.3)	89 (23.2)	196 (51.2)	44 (11.5)	3.58	
I know where to find helpful health resources on the internet	11 (2.9)	50 (13.1)	87 (22.7)	161 (42.0)	74 (19.3)	3.62	
I know how to find helpful health resources on the internet	8 (2.1)	30 (7.8)	111 (29.0)	171 (44.6)	63 (16.4)	3.66	
I know how to use the health information I find on the internet to help me	13 (3.4)	31 (8.1)	94 (24.5)	163 (42.6)	82 (21.4)	3.70	
I know how to use the internet to answer my questions about health	14 (3.7)	30 (7.8)	87 (22.7)	191 (49.9)	61 (15.9)	3.67	
I have the skills I need to evaluate the health resources I find on the internet	12 (3.1)	49 (12.8)	91 (23.8)	165 (43.1)	66( 17.2)	3.58	
I can tell high-quality health resources from low-quality resources	17 (4.4)	36 (9.4)	109 (28.5)	157 (41.0)	64 (16.7)	3.56	
I feel confident in using information from the internet to make health decisions	7 (1.8)	34 (8.9)	64 (16.7)	187 (48.8)	91 (23.8)	3.84	

**Table 3:** eHealth literacy level by background characteristics.

Variables			Low eHealth literacy, n (%)	High eHealth literacy, n (%)
**Sex**				
	Male (n=239)	99 (41.4)		140 (58.6)
	Female (n=144)	59 (41.0)		85 (59.0)
**Age group (years)**				
	20-24 (n=18)	8(44.4)		10 (55.6)
	25-29 (n=254)	106 (41.7)		148 (58.3)
	≥30 (n=111)	44 (39.6)		67 (60.4)
**Professional background**				
	Nurse (n=158)	75(47.5)		83(52.5)
	Medical doctor (n=94)	31 (33.0)		63 (67.0)
	Pharmacist (n=30)	11 (36.7)		19 (63.3)
	Midwife (n=54)	23 (41.8)		32 (58.2)
	Laboratory (n=24)	11 (45.8)		13 (54.2)
	Other (n=23)	7 (31.8)		15 (68.2)
**Work experience (years)**				
	1-3 (n=226)	94 (41.6)		132(58.4)
	4-6 (n=119)	48 (40.3)		71 (59.7)
	≥7 (n=38)	16 (42.1)		22 (57.9)

### Computer Knowledge, Information Retrieval Training Need, and Perception Toward eHealth Information Resources

The majority of the study participants (314/383, 82.0%) stated that they require training in retrieving information from eHealth information sources. Approximately one-third of the participants (131/383, 34.2%) had poor computer knowledge. The majority of the participants (268/383, 70.0%) agreed that eHealth information resources are a useful tool in supporting them in making health-related decisions, and 57.7% (221/383) of the participants also perceived that retrieving information from these sources is easy.

### Factors Associated With eHealth Literacy

Bivariable and multivariable logistic regression analyses were used to discover potential predictor variables linked with eHealth literacy. Variables with *P*<.20 in the bivariable analysis were further considered in the multivariable logistic regression analysis. Finally, the multivariable logistic regression analysis revealed that the variables perceived usefulness, computer access, perceived ease of use of eHealth information resources, and computer knowledge were significantly associated with health professionals’ eHealth literacy; thus, all three hypotheses were supported.

Perceived usefulness was significantly associated with eHealth literacy. Health professionals who perceived using health information resources located on the internet as useful were approximately 2-times more eHealth literate than their counterparts who perceived using eHealth information resources as not useful. Respondents who had computer access were also more than 2-times more likely to be eHealth literate than those who did not have computer access (Table 4).

The computer knowledge of health professionals was also significantly associated with eHealth literacy. Study participants who had good computer knowledge were 2.3-times more eHealth literate than those who had poor computer knowledge (Table 4).

**Table 4:** Multivariable logistic regression analysis of factors associated with eHealth literacy.

Variables		Adjusted odds ratio (95% CI)	*P* value	
**Sex**				
	Male (reference)	1 (1-1)	—^a^	
	Female	1.17 (0.718-2.479)	.52	
**Age group (years)**				
	20-24 (reference)	1 (1-1)	—	
	25-29	0.774 (0.242-2.479)	.68	
	≥30	0.63 (0.162-2.467)	.51	
**Computer access**				
	No (reference)	1 (1-1)	—	
	Yes	2.32 (1.389-3.861)	<.001	
**Work experience (years)**				
	1-3 (reference)	1 (1-1)	—	
	4-6	0.99 (0.529-1.85)	.97	
	≥7	0.97 (0.336-2.79)	.95	
**Computer knowledge**				
	Poor (reference)	1 (1-1)	—	
	Good	2.34 (1.442-3.787)	.001	
**Perceived usefulness**				
	Not useful (reference)	1 (1-1)	—	
	Useful	1.82 (1.075-3.091)	.03	
**Perceived ease of use**				
	Not easy (reference)	1 (1-1)	—	
	Easy	4.53 (2.768-7.401)	<.001	

^a^Not applicable.

## Discussion

### Principal Findings

Higher levels of eHealth literacy may help people make better health-related decisions, resulting in better health outcomes [28]. This study found that the higher the eHealth literacy, the more it will promote social media use for health information, health information–seeking behaviors, and self-care agency. This will lead to better health-promoting behavior by raising the motivation and intention for health promotion [29]. The main purpose of this study was to estimate the level of eHealth literacy and identify its potential predictors among health care providers working in Amhara regional state teaching hospitals in Ethiopia during the COVID-19 pandemic. Our results showed that health professionals in Amhara regional state teaching hospitals have a moderate level of eHealth literacy (mean 29.21). The findings also revealed that computer access, perceived usefulness of eHealth information resources, perceived ease of use of eHealth information resources, and computer knowledge were significantly associated with health professionals’ eHealth literacy level.

### Comparison With Prior Work

In a study on Iranian medical and health science university students, the mean eHEALS score was 28.21 [30]. However, health professionals in Germany have higher eHealth literacy levels [31]. The possible reason for this discrepancy might be due to the variation of internet penetration between these countries, which is 19% in Ethiopia [32] and 93% in Germany [33]. Additionally, due to the limited availability of health-related information in languages other than English, geographical location, cultural, and language barriers may have an impact on eHEALS scores [34,35].

The majority of participants agreed that the internet assisted them in making health-related decisions, and while they believe they know where to find helpful health resources on the internet and how to use them, nearly two-fifth of participants were not confident in their ability to evaluate the information they have retrieved. In particular, the participants’ inability to distinguish between high- and low-quality health resources on the internet suggests a potential weakness in their ability to recognize crucial characteristics that would aid in determining which website may be reliable. Despite the increasing availability of eHealth information and increased acceptance of this mode of communication, all populations, including health professionals, may lack the skills to keep up with this dynamic and changing medium [36].

In terms of professional background, we found that nurses and medical doctors had greater eHealth literacy levels than midwives, pharmacists, and other health professionals. This finding is consistent with a previous study conducted among health care workers in Vietnam [16]. This might be due to the fact that doctors have more professional training than other health professionals, and they have been identified as the group with the greatest capacity for finding, analyzing, justifying, and using health care–related information [37]. Furthermore, because nurses and doctors are the primary caregivers responsible for educating and directing patients, health literacy has been identified as a strategic approach for improving patient–health care worker communication [38].

This study also investigated the factors that can influence eHealth literacy. Computer access, perceived usefulness of eHealth information resources, perceived ease of use, and computer knowledge were identified as significant predictors. Participants who had computer access in their working area were more likely to be eHealth literate than those who did not. Low access to technologies could be the main reason behind the low access to eHealth services [39].

The participants’ eHealth literacy score was associated with their computer knowledge. This finding is consistent with studies that showed a positive association between technology literacy and eHealth literacy [26,40]. A Bangladeshi study also revealed a significant association between eHealth literacy and computer knowledge among university students [25]. Participants who had computer expertise had higher eHealth literacy levels than those who had poor computer knowledge. Because of Ethiopia’s status as a developing country, computer access is limited, literacy skills are insufficient, and health professionals consequently do not have equal access to eHealth resources.

In contrast to other studies [16], demographic variables such as the participants’ sex and age were not found to be significantly associated with eHealth literacy in this study. In a study conducted in Jordan, a nonsignificant association was found between sex, age, and eHealth literacy [22]. Similarly, no significant association was found between gender and eHealth literacy in an Italian study, whereas there was a significant association between age and eHealth literacy [23]. Further studies are needed to investigate the associations between gender, age, and eHealth literacy.

eHealth literacy was also significantly associated with the perceived usefulness of eHealth information sources. Participants who perceived eHealth information resources to be useful for making decisions were more eHealth literate than those who perceived these resources as not being as useful. These findings are supported by studies conducted among nursing students in Jordan [22] and Nepal [24].

It has been suggested that eHealth should be integrated into the health care system, as it can provide certain benefits for improving the quality of health care received [41]. Health professionals should be informed on the latest information and skills to acquire competency in using eHealth resources for patient care and clinical decision-making. According to a preliminary situation assessment, eHealth initiatives in Ethiopia are characterized as being of small scale and unable to effectively communicate with each other (ie, low interoperability). Accordingly, the Ethiopian government developed and formulated a national eHealth strategy for coordinating and streamlining the eHealth initiatives underway in the country as well as for establishing a foundation for sustainable eHealth implementation [42]. When promoting eHealth literacy among health professionals, perceived usefulness and ease of use of eHealth information sources, along with training on information retrieval, computer knowledge, and access should be considered.

### Conclusions

This study provides an overview of health professionals’ eHealth literacy levels in the Amhara regional state, Ethiopia, revealing that more than half of these professionals have a high degree of eHealth literacy. Additionally, the factors associated with eHealth literacy were explored, with the results suggesting significant associations of perceived usefulness, perceived ease of use, computer access, and computer knowledge. To improve health professionals’ eHealth literacy, which could help them assist in decision-making, multidisciplinary approaches are needed. This would help to minimize the risk of infectious diseases such as COVID-19 and further mitigate its impacts. Health professionals also require eHealth literacy to assist their patients in obtaining more up-to-date, reliable, and high-quality information. It is crucial to provide training and support to health care workers on how to find, interpret, and, most importantly, evaluate the quality of health information found on the internet to improve their eHealth literacy level. Further research is needed to explore the role of eHealth literacy for mitigating pandemics in developing countries.

## Abbreviations

eHEALS: eHealth Literacy Scale
UOG: University of Gondar
